# Author Correction: Efficient generative modeling of protein sequences using simple autoregressive models

**DOI:** 10.1038/s41467-022-29593-x

**Published:** 2022-04-01

**Authors:** Jeanne Trinquier, Guido Uguzzoni, Andrea Pagnani, Francesco Zamponi, Martin Weigt

**Affiliations:** 1 Sorbonne Université, CNRS, Institut de Biologie Paris Seine, Biologie Computationnelle et Quantitative LCQB, F-75005 Paris, France; 2 Laboratoire de Physique de l’Ecole Normale Supérieure, ENS, Université PSL, CNRS, Sorbonne Université, Université de Paris, F-75005 Paris, France; 3 Department of Applied Science and Technology (DISAT), Politecnico di Torino, Corso Duca degli Abruzzi 24, I-10129 Torino, Italy; 4 Italian Institute for Genomic Medicine, IRCCS Candiolo, SP-142, I-10060 Candiolo (TO), Italy; 5 INFN Sezione di Torino, Via P. Giuria 1, I-10125 Torino, Italy

**Keywords:** Machine learning, Protein design, Statistical methods, Molecular evolution, Biological physics

Correction to: *Nature Communications* 10.1038/s41467-021-25756-4, published online 04 October 2021.

The original version of this Article contained an error in Fig. 3, in which the predictions presented were run with an incorrect regularization strength. The correct version of Fig. 3 is:
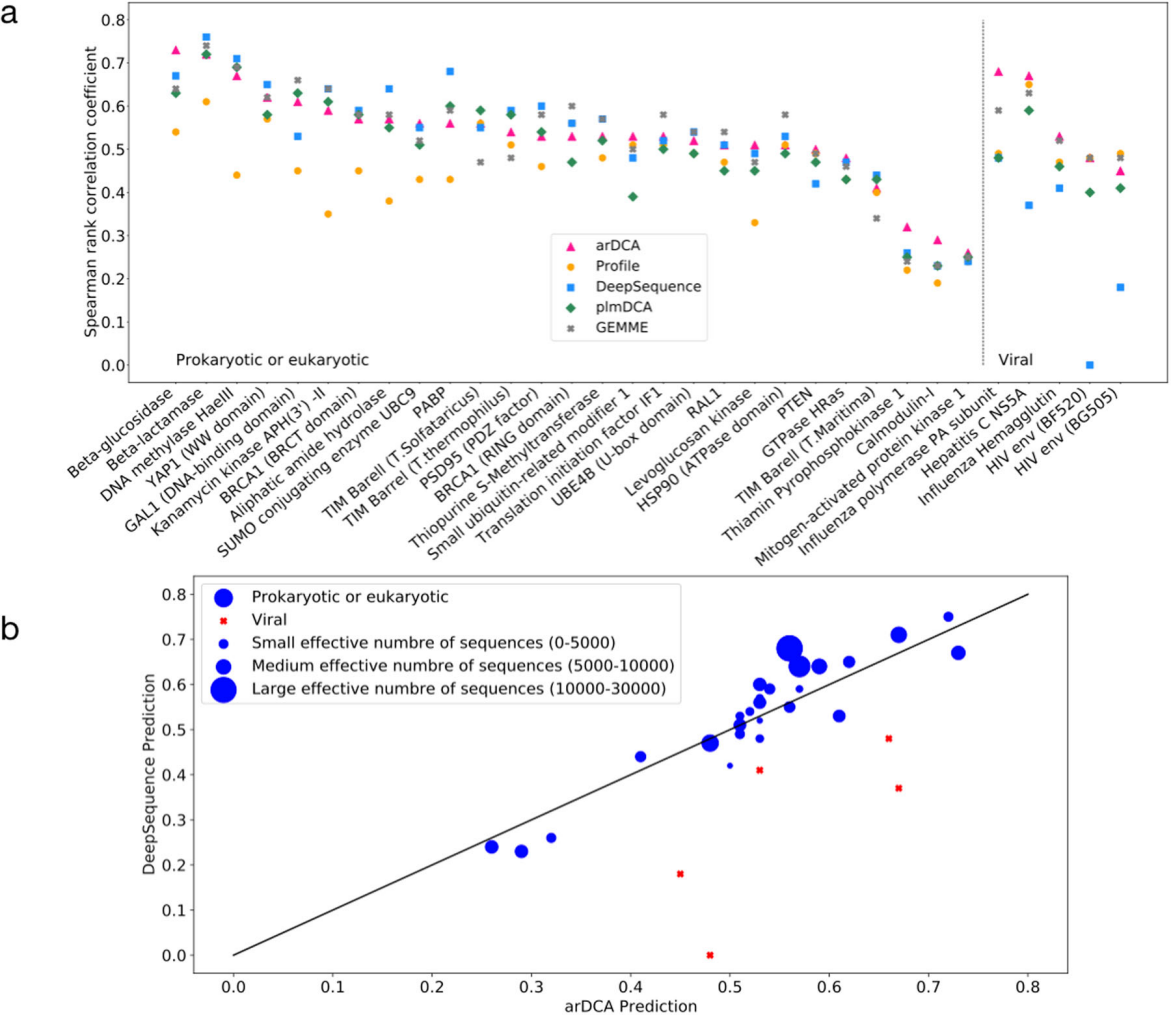


which replaces the previous incorrect version:
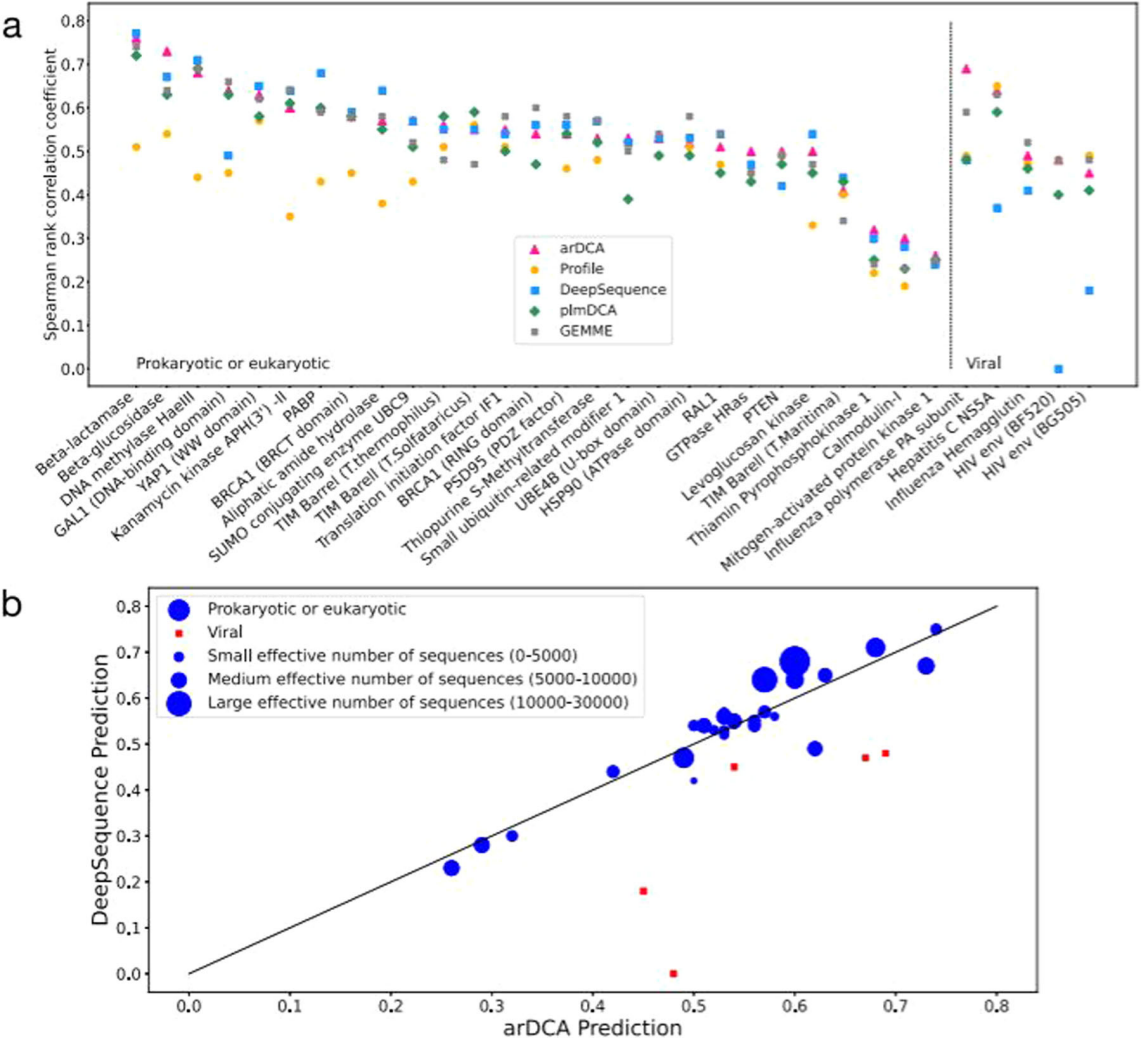


In addition, the sentence “Almost all dots are close to the diagonal (apart from few viral datasets), with 15/32 datasets having a better arDCA prediction and 17/32 giving an advantage to DeepSequence.” has been corrected to “Almost all dots are close to the diagonal (apart from few viral datasets), with 17/32 datasets having a better arDCA prediction and 15/32 giving an advantage to DeepSequence”. The sentence “We also add an L2 regularization, with a regularization strength of 0.0001 for the generative tests and 0.01 for mutational effects and contact prediction.” has been corrected to “We also add an L2 regularization, with a regularization strength of ${\lambda }_{J}={{{{1}}}}{{{{{0}}}}}^{{{{{-}}}}{{{{4}}}}}{{{{,}}}}\,{{{{{\lambda }}}}}_{{{{{ h}}}}}={{{{1}}}}{{{{{0}}}}}^{{{{{-}}}}{{{{6}}}}}$ for the generative tests and ${\lambda }_{J}={{{{1}}}}{{{{{0}}}}}^{{{{{-}}}}{{{{2}}}}}{{{{,}}}}\,{{{{{\lambda }}}}}_{{{{{h}}}}}={{{{1}}}}{{{{{0}}}}}^{{{{{-}}}}{{{{4}}}}}$ for mutational effects and contact prediction.”

This has been corrected in both the PDF and HTML versions of the Article.

