# Supplementary material for: Efficient generative modeling of protein sequences using simple autoregressive models
Source: Nat Commun. 2021 Oct 4;12:5800. doi: 10.1038/s41467-021-25756-4 (PMC8490405; doi:10.1038/s41467-021-25756-4)
Supplement: Supplementary file 1 — Supplementary Information [file 41467_2021_25756_MOESM1_ESM.pdf]

Supplementary information: Efficient generative modeling of protein sequences using simple autoregressive models

Jeanne Trinquier,[1,2] Guido Uguzzoni,[3,4] Andrea Pagnani,[3,4,5] Francesco Zamponi,[2] and Martin Weigt[1]

[1] *Sorbonne Université, CNRS, Institut de Biologie Paris Seine, Biologie Computationnelle et Quantitative LCQB, F-75005 Paris, France*

[2] *Laboratoire de Physique de l'Ecole Normale Supérieure, ENS, Université PSL, CNRS, Sorbonne Université, Université de Paris, F-75005 Paris, France*

[3] *Department of Applied Science and Technology (DISAT), Politecnico di Torino, Corso Duca degli Abruzzi 24, I-10129 Torino, Italy*

[4] *Italian Institute for Genomic Medicine, IRCCS Candiolo, SP-142, I-10060 Candiolo (TO) - Italy*

[5] *INFN Sezione di Torino, Via P. Giuria 1, I-10125 Torino, Italy*

Supplementary Note 1. POSITIONAL ORDER

For a family of length L, there are $L!$ possible permutations of the sites and therefore $L!$ possible orders. The parameterization of the conditional probabilities of the arDCA model is not invariant under a change of order, thus different orders may give different results. However, an optimization over all the different orders is not computationally feasible. We compared some particular orders: the direct order along the protein chain, the entropic order where the sites are ordered in ascending order according to their local entropy $s_i = -\sum_{a=1}^{q} f_i(a) \log f_i(a)$, and the inverse entropic order where s_i is used in descending order. A comparison with 100 random orders was also made. The quality of the generative properties was found to be highly correlated with the log-likelihood of the optimized model, which can be computed exactly after the parameters are inferred, see Section Methods of the main text. Supplementary Figure 1 shows a comparison of the likelihood and the Pearson's correlation of the two-point statistics for the different orders. The values reported for the random order is an average over the 100 different realizations, with one standard deviation given by the vertical bar. While the direct order is compatible with a random order, for all but one family the entropic order has the highest value of the likelihood and maximizes the Pearson correlation.

In order to check that the entropic order is a good heuristic choice between all possible orders, we designed a greedy procedure to increase the likelihood by doing some permutations between the sites. This procedure tries to find a locally optimal order. The different steps of the procedure are:

- Choose a site randomly
- Permute this site with all the other sites and compute the likelihood of the new model each time
- Choose the permutation that increases the most the likelihood and iterate the procedure

Supplementary Figure 2a shows the evolution of the log-likelihood of the entropic and direct orders of the family PF00014 under permutations. The permuted entropic order saturates quickly to a value of the log-likelihood relatively close to the initial one, indicating that the entropic order is not far from a locally optimal one. The direct order saturates to the same value of the log-likelihood. The plot also shows the evolution of the Kendall-Tau distance between the two orders, defined as the number of pairs in a different order, i.e. for two lists $l1$ and $l2$,

$$K(l1, l2) = |\{(i, j) : i < j, (l1(i) < l1(j) \cap l2(i) > l2(j)) \cup (l1(i) > l1(j) \cap l2(i) < l2(j))\}| \ . \quad \text{(S1)}$$

The Kendall-Tau distance gives a measure of the dissimilarity between two lists. Supplementary Figure 2a shows that the distance between the two orders decreases with increasing permutations. Supplementary Figures 2b and 2c show the value of the local entropy for each site in both orders before (b) and after (c) 60 permutations. After 60 permutations, it is clear that the sites with a low local entropy are typically at the beginning, which is coherent with the explanation given in the main text.

Finally, Supplementary Figure 3 shows the Pearson correlation of two-point connected correlations for the 33 families used for mutational effects with the entropic and the direct order. Coherently with the previous discussion, the entropic order gives a better result for 30 over 33 families.

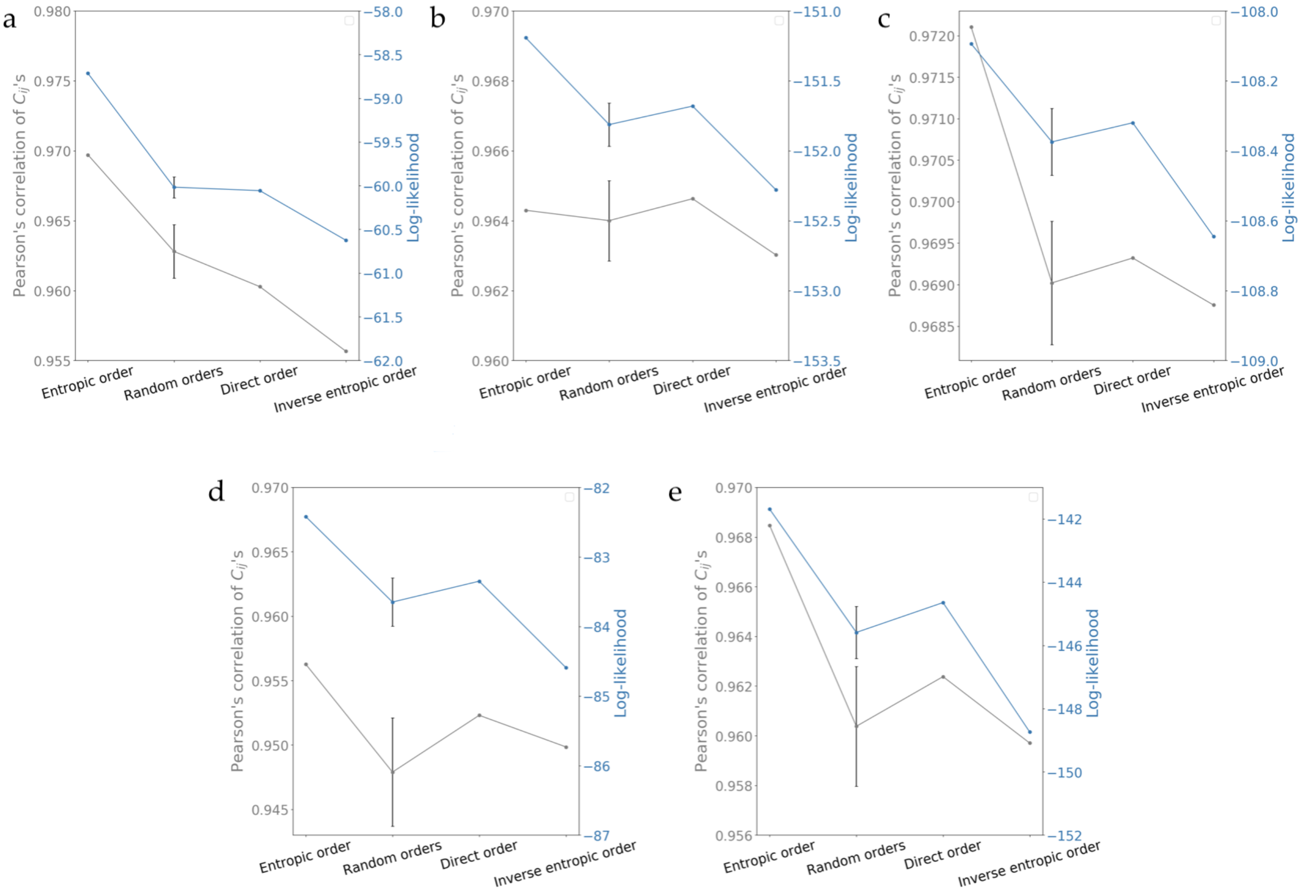

Supplementary Figure 1. Comparison of the Pearson correlation of two-point connected correlations and the log-likelihood between different orders. The value of the random order is the mean of 100 random orders with one standard deviation given as a vertical bar. Results are for Pfam families PF00014 (a), PF00072 (b), PF00076 (c), PF0595 (d), and PF13354 (e).

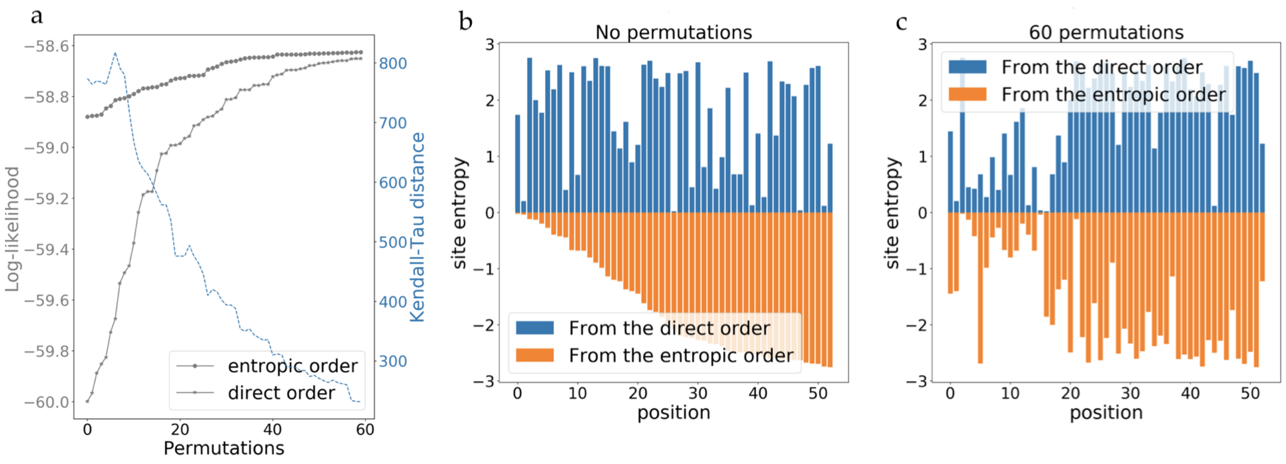

Supplementary Figure 2. a: Evolution of the log-likelihood and the Kendall-Tau distance of the direct and entropic orders under permutations. b and c: Values of the entropy of each site in the direct and entropic orders with no permutations (b) and after 60 permutations (c).

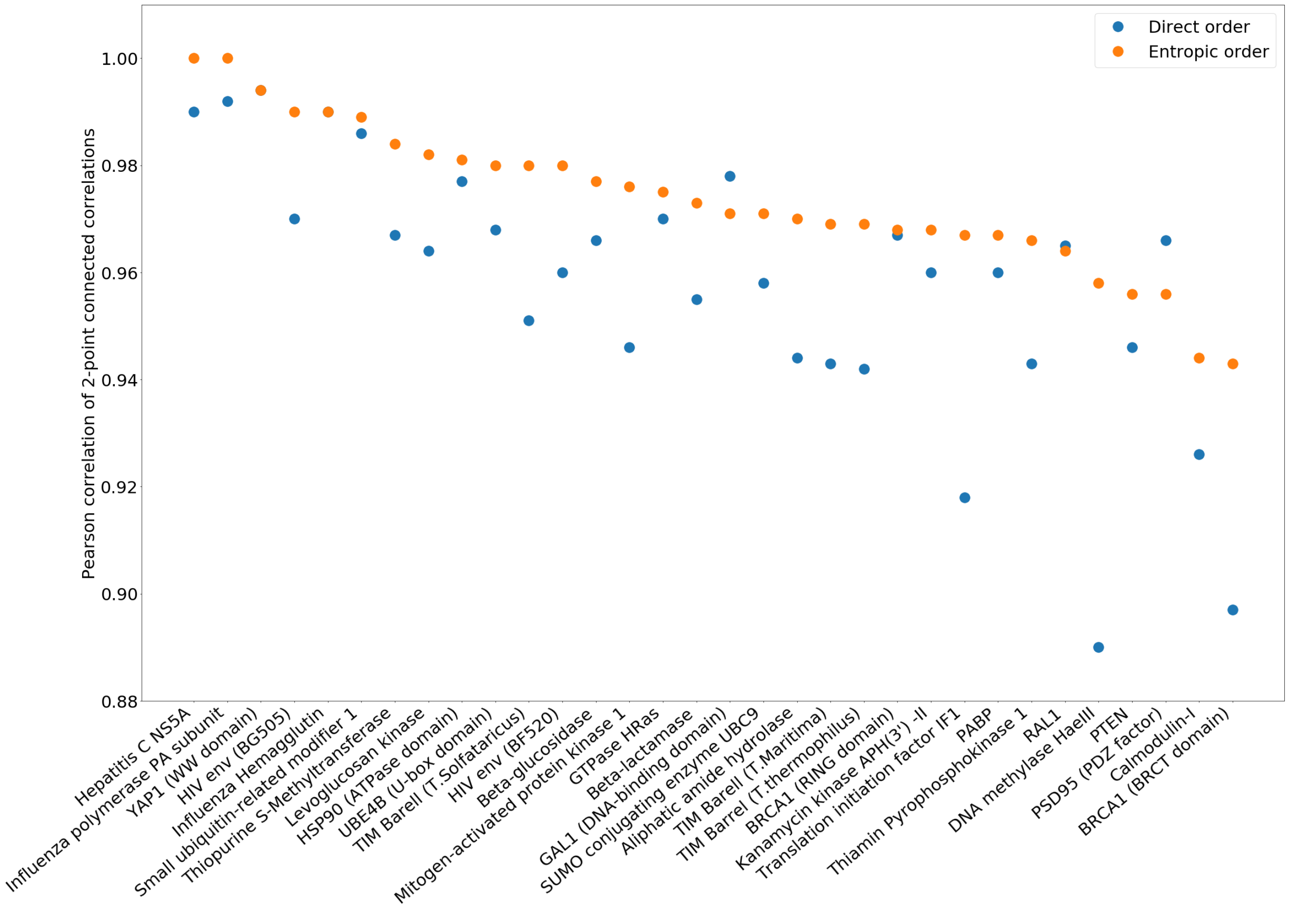

Supplementary Figure 3. Pearson correlation of the two-point connected correlations for all the 33 families used for mutational effects, for the direct and entropic orders.

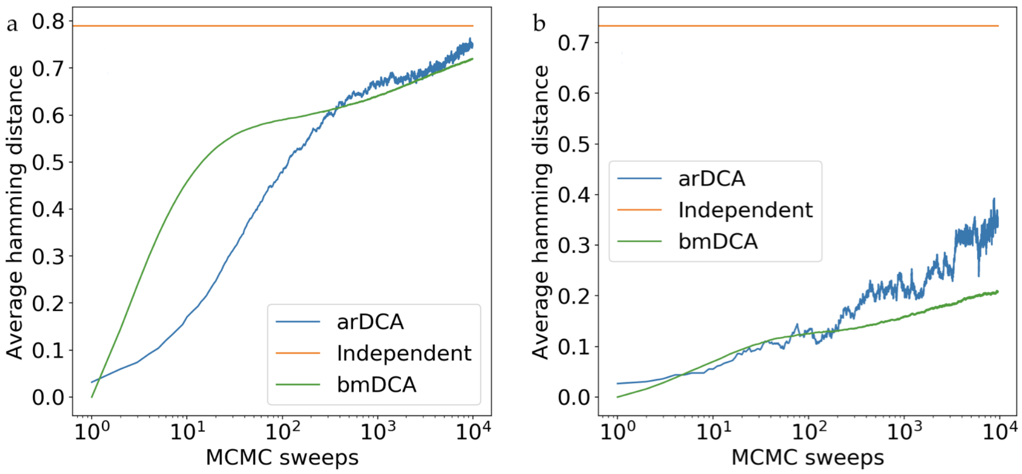

Supplementary Figure 4. Averaged Hamming distance between a sequence and its time evolution after MCMC sweeps, in bmDCA and in arDCA. The average is made with respect to 100 (panel a, PF13354 family) or 5 (panel b, PF18589 family) initially equilibrated sequences. The horizontal line is the average Hamming distance between two independent equilibrium sequences, which by the direct arDCA-sampling procedure can be achieved in the equivalent of a single sweep (each positions sampled once).

Supplementary Note 2. SAMPLING FROM THE MODEL

To emphasize the advantages of the direct sampling protocol of arDCA, we report here a comparison with sampling from the bmDCA model. In this case, sequences must be obtained via MCMC sampling, but a lot of moves have to be made in order to achieve a proper equilibration in some families [1, 4, 5]. Furthermore, several hyperparameters have to be set, such as the number of MCMC independent chains, the total length and the number of samples produced by each chain, etc. As discussed in detail in [4], these hyperparameters heavily affect the quality of the training and sampling in bmDCA. If the mixing time of MCMC is short enough, then it is possible to train and sample the bmDCA model in equilibrium, which leads to stable and reproducible results. If the mixing time is too long, however, this becomes impossible and one is forced to train the machine out-of-equilibrium (e.g. via CD or PCD), which leads to an unstable resampling displaying a maximal quality at some given sampling time that depends on the training history [4].

As an illustration of these effects, we consider a beta-lactamase family (Pfam family PF13354), which is particularly hard to sample via MCMC; bmDCA models have then been trained via PCD [1]. In order to directly compare with MCMC sampling of bmDCA, we sampled sequences from our arDCA model, using a Metropolis-Hasting procedure. We propose a random change of a residue, and we accept the move with a probability that depends on the ratio of the probabilities of the new and old sequences. This is of course a very inefficient way of sampling from the arDCA model, but it allows for a direct comparison with the MCMC dynamics of a bmDCA. The Hamming distance between an initial equilibrium sequence (obtained via the sequential procedure described above, which thus guarantees equilibration) and its time evolution after MCMC sweeps was computed. This time-dependent Hamming distance, averaged over 100 initial sequences, is reported in Supplementary Figure 4a. Its shape is very similar to that obtained by MCMC sampling of bmDCA [1], also reported in the same figure (note that in this case the initial sequence is not fully equilibrated). It grows very slowly with time, and only at very long times it saturates to the equilibrium Hamming distance between two independently sampled sequences. The time it takes to reach this plateau gives an estimation of the number of MCMC sweeps needed to obtain an equilibrium sample. Supplementary Figure 4a shows that the equilibration takes at least 10^4 MCMC sweeps. On the other hand, the sequential procedure described above, which is only possible for arDCA models, allows one to sample almost instantaneously, thus completely bypassing the long time scale associated to MCMC.

We repeated the same study for the obesity receptor family (ObR_IG, Pfam family PF18589) and for the Leptin family (Pfam family PF02024), where the number of available sequences is more limited and bmDCA has shown some convergence problems. We find that the mixing time of MCMC is even larger in that case, see Supplementary Figure 4b for PF18589. As a result, the bmDCA training is strongly out-of-equilibrium. While the Pearson coefficient of the C_{ij} reaches 0.99 during training, a resampling of the same model leads to very poor results (~ 0.62 for PF18589 and ~ 0.43 for PF02024). We conclude that bmDCA, at least in our simplest scheme, is not reliable. On the contrary,

arDCA provides reliable results for both families.

We note that these observations have interesting implication for the problem of sampling in disordered systems with slow dynamics, as already noted in [9].

Supplementary Note 3. RESULTS FOR OTHER FAMILIES

A. Pfam Datasets

1. *Description*

We describe the properties of the five Pfam families used to test the generative properties and structure prediction of the arDCA model: PF00014, PF00072, PF00076, PF00595, PF13354. MSA are downloaded from Pfam (http://pfam.xfam.org/) and sequences with more than 6 consecutive gaps are removed. The value of M_{eff} defined in Section Methods of the main text gives the effective number of sequences, obtained by a proper reweighing of very similar sequences.

Pfam identifier	PF00014	PF00072	PF00076	PF00595	PF13354
Protein domain	Kunitz domain	Response regulator receiver domain	RNA recognition motif	PDZ domain	Beta-lactamase
L	53	112	70	82	202
M	13600	823798	137605	36690	7515
M_{eff}	4364	229585	27785	3961	7454

Supplementary Table 1. Properties of the Pfam families

2. *Principal component analysis*

Supplementary Figure 5 shows the projection of natural sequences (first column), sequences sampled from arDCA (second column), bmDCA (third column) and the profile model (last column) in a two-dimensional space, constructed by performing principal component analysis on the natural sequences. Each bin in the figure has a color related to its total weight, defined by resampled the sequences using the weights defined in Section Methods of the main text.

3. *Frequencies*

Supplementary Figure 6 shows how well the model is able to reproduce the empirical frequencies obtained from the data. The one-point frequencies (left), two-point (center) and three-point (right) connected correlations are shown, both from the arDCA model (blue) and the bmDCA (red). Note that for the three-point connected correlations, the correlations that have an empirical value smaller than 0.003 are removed, because they are not meaningful given the limited number of sequences in the dataset.

B. Families used for mutational effects

We show in Supplementary Table 2 the generative properties of the arDCA model for the 33 families that are used for mutational effect predictions [6, 7]. The computational time of parameter learning on a standard laptop is also included.

C. Comparison of mutational predictions of DeepSequence and arDCA

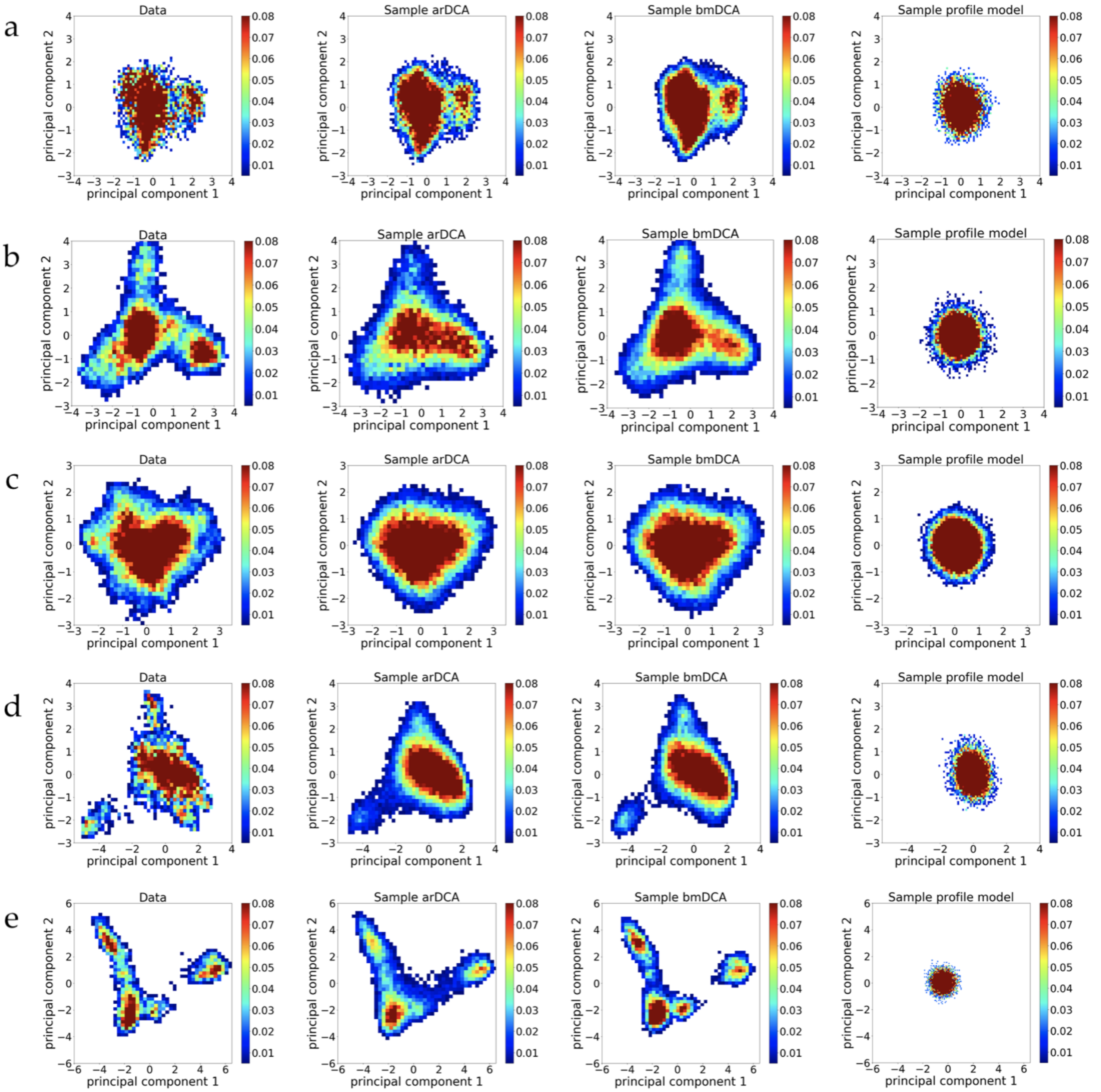

Supplementary Figure 5. Projections of sequences on the principal components obtained from natural sequences, for the Pfam families PF00014 (a), PF00072 (b), PF00076 (c), PF0595 (d), and PF13354 (e).

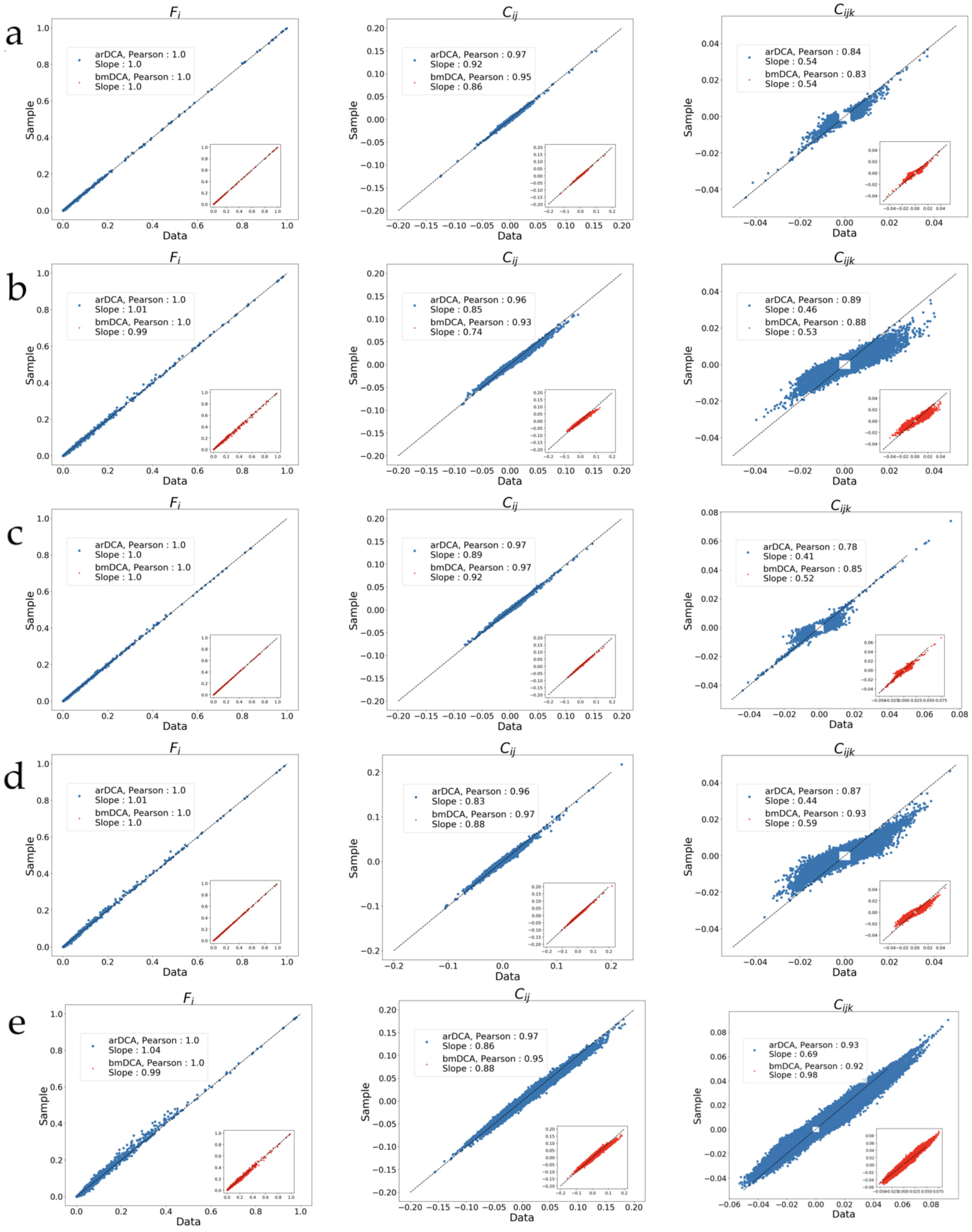

Supplementary Figure 6. One-point frequencies and two- and three-point connected correlations, obtained from resampling the models (vertical axis) and from empirical data (horizontal axis) for the Pfam families PF00014 (a), PF00072 (b), PF00076 (c), PF0595 (d), and PF13354 (e).

Family	L	M	M_{eff}	Time (min)	Pearson's correlation of C_{ij}
YAP1 (WW domain)	30	85299	5822	4	0.99
GAL1 (DNA-binding domain)	62	20688	6435	5	0.98
Translation initiation factor IF1	69	9090	1310	2	0.98
RAL1	71	33026	6435	9	0.97
BRCA1 (RING domain)	75	39396	6585	12	0.96
UBE4B (U-box domain)	75	16478	2941	5	0.98
Small ubiquitin-related modifier 1	76	21695	2669	5	0.99
PABP	79	246405	29045	77	0.97
PSD95 (PDZ factor)	82	208112	7215	60	0.97
Hepatitis C NS5A	113	11423	55	4	1
SUMO conjugating enzyme UBC9	138	32486	4957	37	0.97
Calmodulin-I	139	36224	7196	30	0.94
GTPase HRas	164	84762	12506	130	0.98
Thiopurine S-Methyltransferase	177	6688	2351	13	0.98
BRCA1 (BRCT domain)	186	8391	2037	14	0.94
Thiamin Pyrophosphokinase 1	201	9966	3851	26	0.98
HSP90 (ATPase domain)	218	23447	2847	40	0.98
Kanamycin kinase APH(3')-II	226	29808	9658	60	0.96
TIM Barrel (T.thermophilus)	236	23742	4869	98	0.97
TIM Barrel (T.Solfataricus)	237	23743	4913	101	0.97
TIM Barrel (T.Maritima)	239	23745	5001	103	0.97
Aliphatic amide hydrolase	247	76372	20145	340	0.97
Beta-lactamase	252	14783	3818	60	0.97
Mitogen-activated protein kinase 1	288	65626	7322	400	0.98
PTEN	304	8566	1119	43	0.96
DNA methylase HaeIII	318	26513	11098	230	0.96
Levoglucosan kinase	364	12925	3638	160	0.98
Beta-glucosidase	441	49471	8477	400	0.98
Influenza Hemagglutin	544	51000	62	500	0.99
HIV env (BF520)	657	73441	305	800	0.98
Influenza polymerase PA subunit	716	19611	9	518	1

Supplementary Table 2. MSA parameters, running time and Pearson correlation for C_{ij} for the 32 families used for comparison with DMS data.

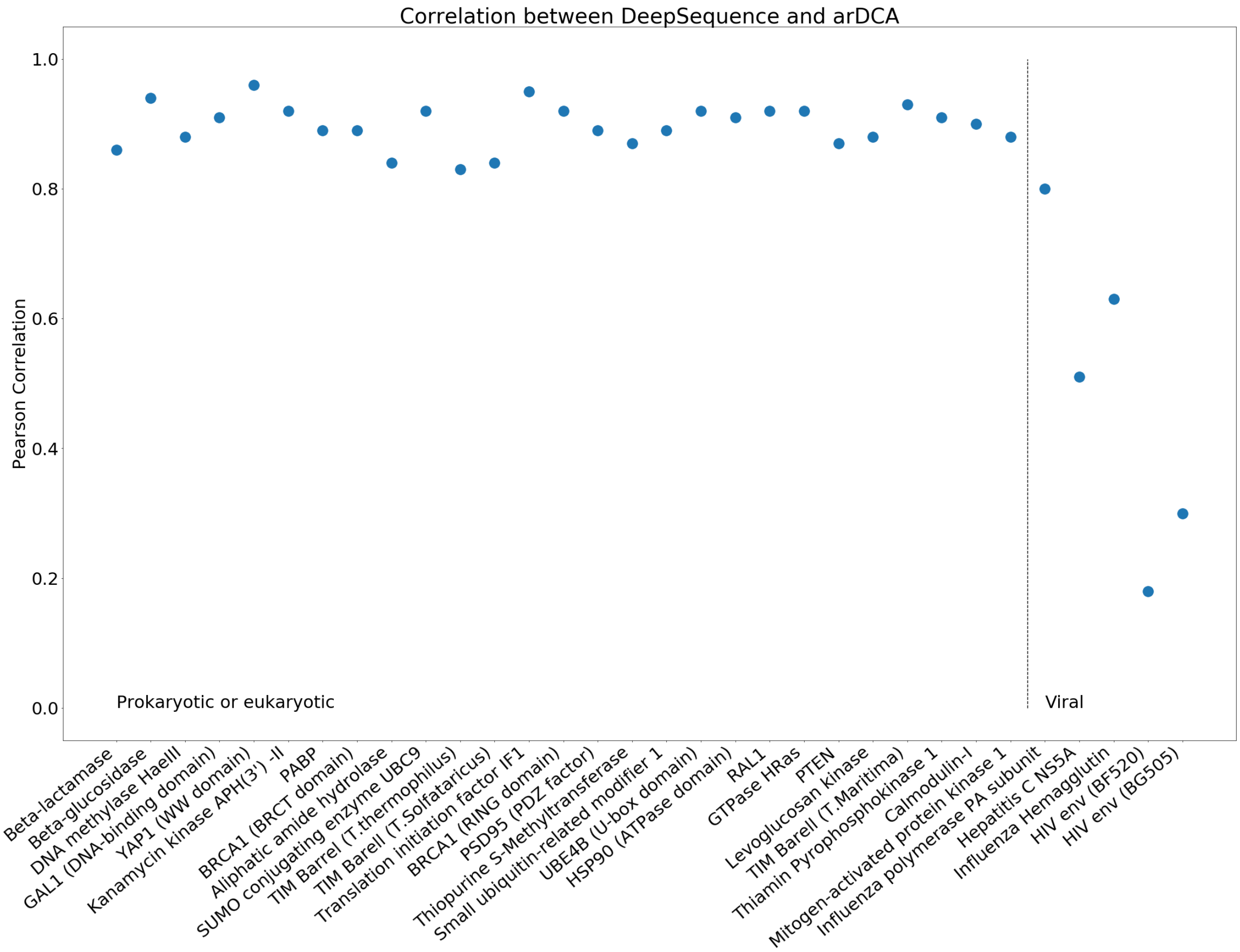

Supplementary Figure 7. Pearson correlations for mutational predictions of arDCA and DeepSequence across all protein families studied.

Supplementary Note 4. CONTACT PREDICTION

Once the effective couplings are calculated, the standard procedure of DCA is applied [3]. First, each pair of amino acids is assigned an interaction score given by the Frobenius norm:

$$F_{ij} = \|\boldsymbol{J}_{ij}\|^2 = \sqrt{\sum_{a,b=1}^{20} J_{ij}(a,b)^2} \ . \tag{S2}$$

Note that the gap state ($q = 21$) is not taken into account in the norm. Because of overparametrization caused by the non-independence of the empirical frequencies, both the Potts model and autoregressive models are invariant under some gauge transformations, that is different sets of parameters give the same probability. On the other side, the Frobenius norm is not invariant by gauge transformation, so a gauge choice is needed. The zero-sum gauge was found to be the gauge that minimizes the Frobenius norm; in other words, this gauge choice includes in the couplings only the information that cannot be treated by fields. Note that the zero-sum gauge is also the gauge of the standard Ising model. The equations characterizing the zero-sum gauge are: $\sum_a h_i(a) = \sum_a J_{ij}(a,b) = \sum_b J_{ij}(a,b) = 0$. Finally, the so-called average product correction (APC) is applied on the Frobenius scores, because it was empirically shown to improve contact prediction: $F_{ij}^{APC} = F_{ij} - \frac{F_{.j}F_{i.}}{F_{..}}$ where the dot represents the average with respect to the index.

1. *Results across families*

a. PPV – To test the contact prediction, a distance $d(i,j)$ between each heavy atoms in the amino acids was extracted from the crystal structures present in the PDB database. Sites with atoms at a distance < 8 Å were considered in contact. Note that 8 Å is too large to be a true contact, but since we are looking for consensus contacts in the family and there is variability from protein to protein, this definition has become standard. Coherently with the literature standard, a minimal separation of $|i - j| \geq 5$ along the protein chain was imposed in order to consider only non-trivial contacts corresponding to sites that are not close in the chain. Pairs ij are ranked according to the APC-corrected Frobenius norm, as defined in Supplementary Note 4. Supplementary Figure 8 shows the positive predictive value as a function of the number of predicted non-trivial contacts for the arDCA model (blue) and bmDCA (orange). The Positive Predicted Value (PPV) is the fraction of true contacts among the first n predictions, corresponding to the n highest scores.

b. Contact map – Supplementary Figure 10 show the contact maps of the arDCA and bmDCA models. The black crosses represent the true contact map with a threshold of 8 Å. The blue dots are the true positive predictions and the red ones are the false positive considering the $2L$ top predictions of non-trivial contacts.

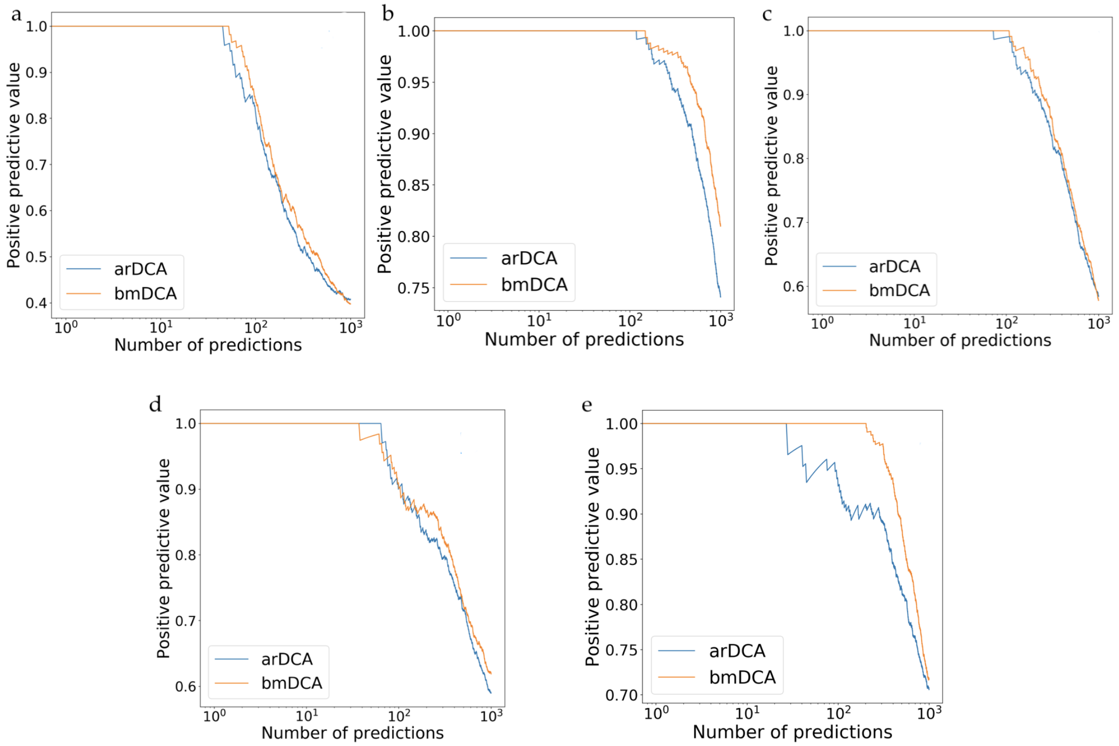

Supplementary Figure 8. PPV curves for the Pfam families PF00014 (a), PF00072 (b), PF00076 (c), PF0595 (d), and PF13354 (e) obtained with arDCA and bmDCA.

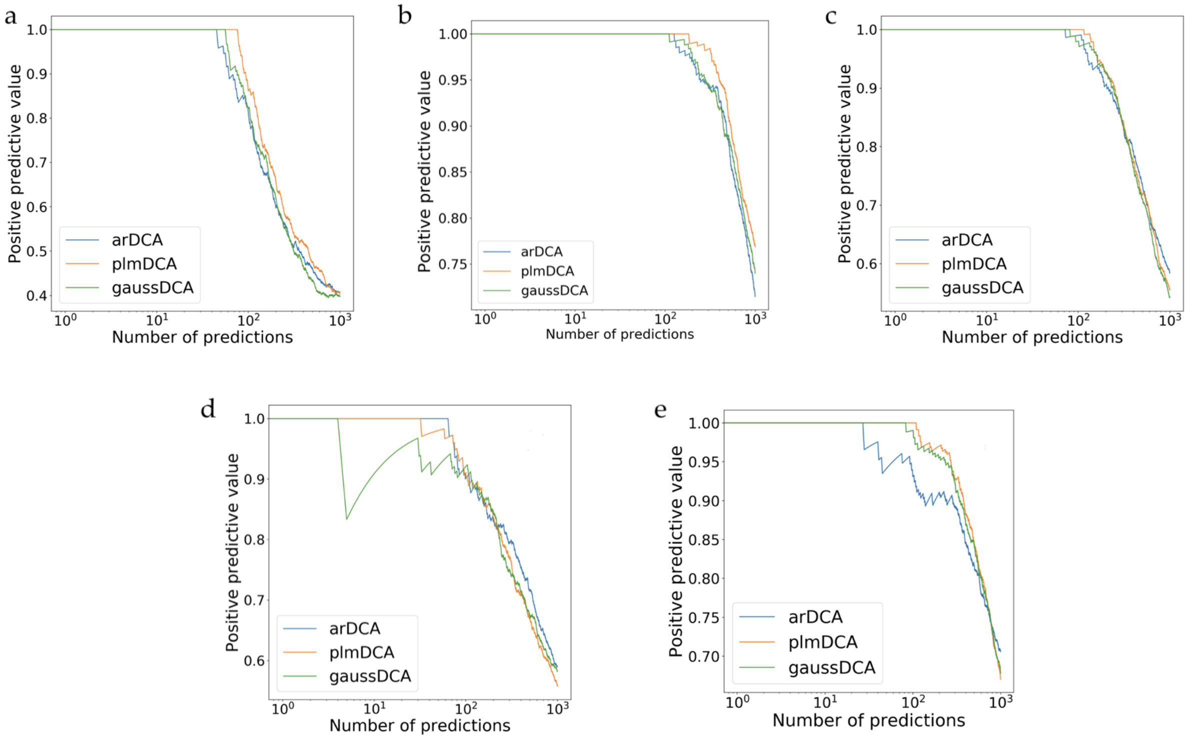

Supplementary Figure 9. PPV curves for the Pfam families PF00014 (a), PF00072 (b), PF00076 (c), PF0595 (d), and PF13354 (e) obtained with arDCA, plmDCA and GaussDCA (aka mfDCA).

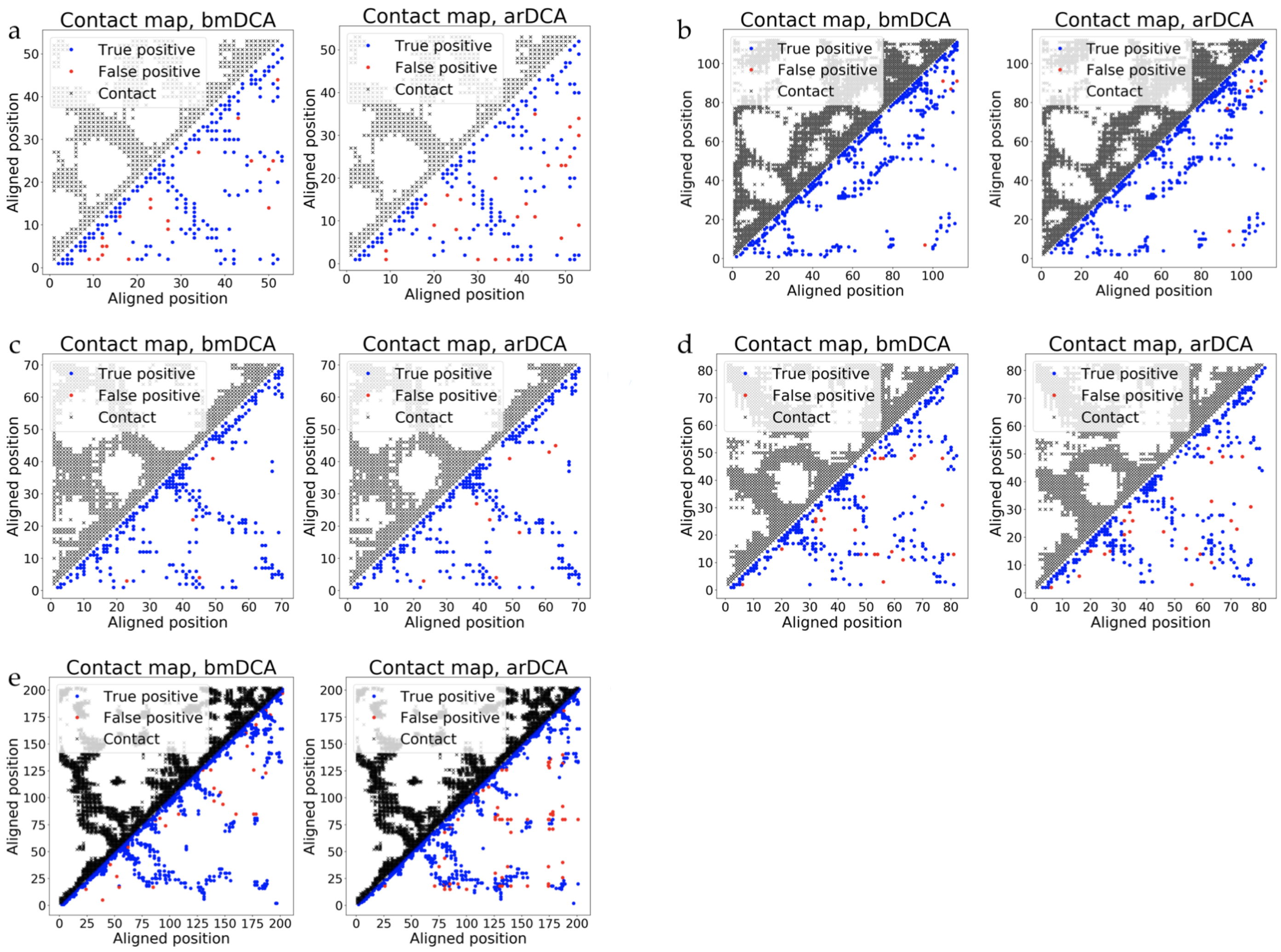

Supplementary Figure 10. Contact maps for the Pfam families PF00014 (a), PF00072 (b), PF00076 (c), PF0595 (d), and PF13354 (e).

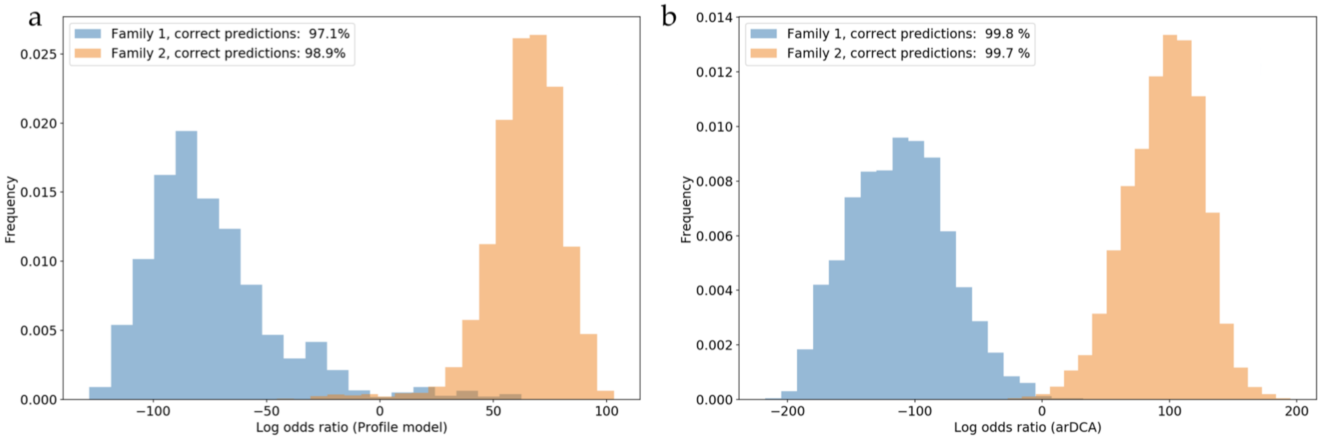

Supplementary Figure 11. Histograms of log-odds rations $\log\{P_1(seq)/P_2(seq)\}$ for subfamily specific profile (panel a) and arDCA (panel b) models, for the two sub-families of the response-regulator domain family (PF00072) identified by the coexistence with one of two DNA-binding domains Trans_reg_C (PF00486) or GerE (PF00196). Models are learned on randomly extracted training sets of 6000 sequences per sub-family, histograms show the test sets (blue for coexistence with PF00486, orange with PF00196). The part of the blue (resp. orange) histograms with negative (resp. positive) values corresponds to correct sub-family annotations via the log-odds ratio; the fraction of correct predictions for each subfamily and each model is indicated in the legend.

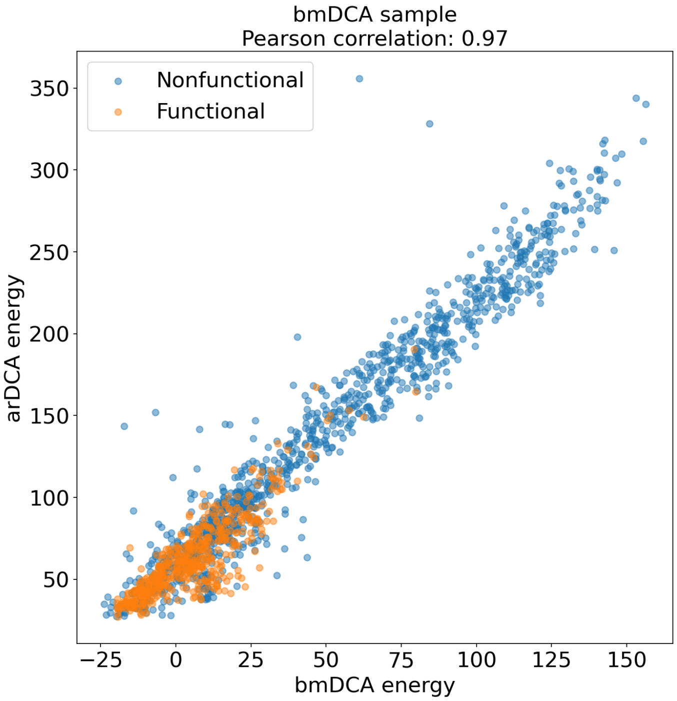

Supplementary Figure 12. Scatter plot of statistical energies of bmDCA and arDCA for the artificial chorismate mutase enzymes enzymes designed in [8]; the bmDCA energies are the ones published along with the references. The coloring allows to distinguish experimentally verified functional (red) and non-functional (blue) sequences. Both energies are highly correlated (Pearson correlation 0.97), and functional sequences are found at low energies only, in perfect agreement with [8].

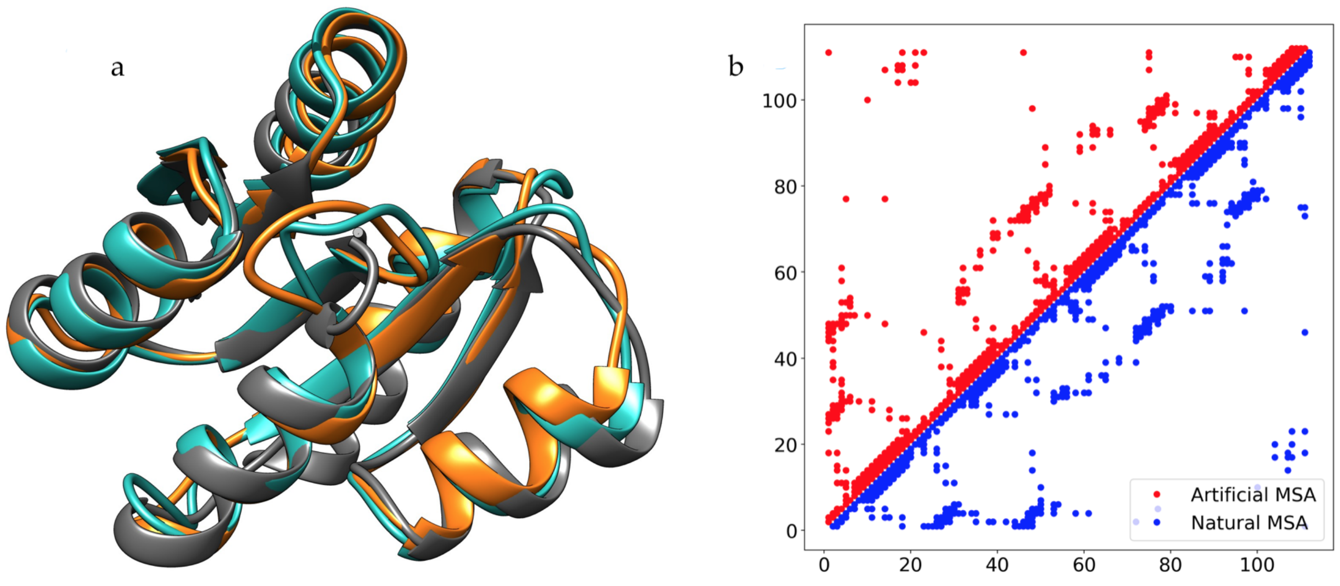

Supplementary Figure 13. Panel a: Comparison of an exemplary PDB structure of the PF00072 family (PDB ID 1nxs [2], grey) with trRosetta predictions for small MSA of 10 arDCA-generated sequences (turquoise, RMSD 1.96Å, and 0.91Å over 94/106 residues with 2Å) and of 10 natural sequences (orange, RMSD 1.74Å, and 0.91Å over 90/108 residues with 2Å). Panel b shows the contact maps for the two trRosetta predictions.

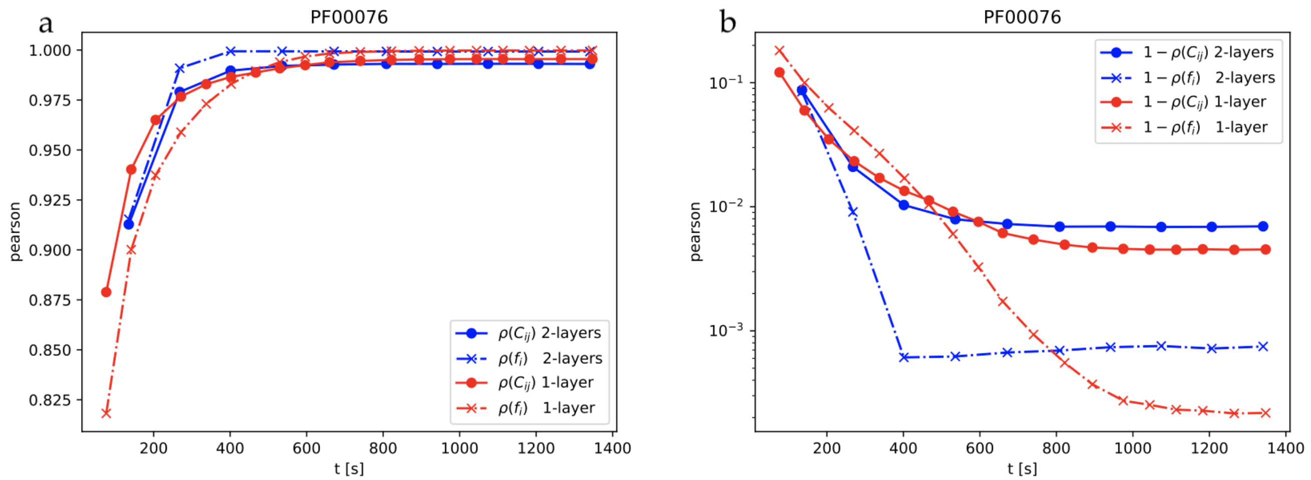

Supplementary Figure 14. Evolution of the Pearson correlation (for one- and two-point statistics) during the learning for the family PF00076. The computational time is given in seconds. Both figures compare the one-layer (red) and the two-layer (blue) models. Figure a shows the Pearson correlation while figure b shows one minus the Pearson correlation (in semilog scale).

Supplementary Note 5. TWO-LAYER AUTOREGRESSIVE MODELS

Due to the very simple structure of the one-layer arDCA model, one might ask whether a more complicated and flexible model could perform better. To address this question, we considered an arDCA model for the family PF00076, but with two layers instead of one. As an exploratory step, we just considered very simple two-layer architectures: each conditional probability $P(a_i|a_{i-1},\ldots,a_1)$, $i \in 2,\ldots,L$ is modeled in terms of a dense input node of size $kq \times (i-1)q$ for (input amino acid sequences are one-hot-encoded) with non a linear activation function σ, while the second layer is again a dense node of size $q \times kq$ concatenated with a *softmax* to get a probability as final output:

$$\tilde{P}_i^{(2-layer)}(a_1,\ldots,a_{i-1}) = \text{softmax}\left(W_2^{[q,kq]} \cdot \sigma\left(W_1^{[kq,(i-1)q]} \cdot \vec{a} + \vec{b}_1^{[kq]}\right) + \vec{b}_2^{[q]}\right) \quad , \quad i \in \{2,\ldots,L\} \quad , \tag{S3}$$

where $W_{1,2}^{[l,m]}$ are parameter matrices of size $l \times m$ and $\vec{b}_{1,2}^{[l]}$ are vectors of parameters (biases) of size l that are optimized in the training step. We tried different values of k, and different types of activation functions σ and we opted for $k = 5$ and $\sigma = \text{ReLU}$.

Supplementary Figure 14 shows that increasing the complexity of the model does not improve the ability to reproduce the statistics of the data. In the specific case of the family PF00076, the one-layer model is even marginally better, both for the one-point and two-point statistics. Moreover, the computational time is comparable in the two cases, therefore using a two-layer model gives neither an advantage on the generative qualities, nor on the computational time.

SUPPLEMENTARY REFERENCES

[1] P. Barrat-Charlaix, A. P. Muntoni, K. Shimagaki, M. Weigt, and F. Zamponi. Sparse generative modeling via parameter reduction of Boltzmann machines: Application to protein-sequence families. *Physical Review* E104:024407 (2021).

[2] C. J. Bent, N. W. Isaacs, T. J. Mitchell, and A. Riboldi-Tunnicliffe. Crystal structure of the response regulator 02 receiver domain, the essential yycf two-component system of streptococcus pneumoniae in both complexed and native states. *Journal of Bacteriology*, 186(9):2872–2879 (2004).

[3] S. Cocco, C. Feinauer, M. Figliuzzi, R. Monasson, and M. Weigt. Inverse statistical physics of protein sequences: a key issues review. *Reports on Progress in Physics*, 81(3):032601 (2018).

[4] A. Decelle, C. Furtlehner, and B. Seoane. Equilibrium and non-equilibrium regimes in the learning of restricted boltzmann machines. *arXiv preprint arXiv:2105.13889* (2021).
[5] M. Figliuzzi, P. Barrat-Charlaix, and M. Weigt. How pairwise coevolutionary models capture the collective residue variability in proteins? *Molecular Biology and Evolution*, 35(4):1018–1027 (2018).
[6] E. Laine, Y. Karami, and A. Carbone. Gemme: a simple and fast global epistatic model predicting mutational effects. *Molecular Biology and Evolution*, 36(11):2604–2619 (2019).
[7] A. J. Riesselman, J. B. Ingraham, and D. S. Marks. Deep generative models of genetic variation capture the effects of mutations. *Nature Methods*, 15(10):816–822 (2018).
[8] W. P. Russ, M. Figliuzzi, C. Stocker, P. Barrat-Charlaix, M. Socolich, P. Kast, D. Hilvert, R. Monasson, S. Cocco, M. Weigt, et al. An evolution-based model for designing chorismate mutase enzymes. *Science*, 369(6502):440–445 (2020).
[9] D. Wu, L. Wang, and P. Zhang. Solving statistical mechanics using variational autoregressive networks. *Physical Review Letters*, 122(8):080602 (2019).
